# Strategies for Coping With the End of a Desirable Intimate Relationship: An Exploratory Study

**DOI:** 10.1177/14747049251368249

**Published:** 2025-08-11

**Authors:** Menelaos Apostolou, Maria Hadjiantoni, Timo Juhani Lajunen

**Affiliations:** 1 Department of Social Sciences, 121343 University of Nicosia, Nicosia, Cyprus; 2 Department of Psychology, 8018 Norwegean University of Science and Technology, Trondheim, Norway

**Keywords:** strategies for coping with a breakup, end of an intimate relationship, coping strategies, breaking up, mating strategies, mating

## Abstract

Frequently, people find themselves in a situation where an intimate relationship they wish to keep ends, creating emotional pain that requires coping strategies. The aim of the present study is to investigate the various strategies people employ for this purpose. Using a mixed-methods approach, we identified 84 distinct acts, which we classified into 16 strategies and subsequently classified into five main strategies for coping with the end of a desirable intimate relationship. The most likely to be used main strategy was “Transfer focus to different things,” including strategies such as “Focus on myself” and “Keep myself busy.” Other commonly chosen main strategies were “Seek support,” involving reliance on friends, family, and professionals, and “Social withdrawal,” characterized by isolation behaviors. Additionally, several participants indicated readiness to adopt the “Disassociation and positive reframing” main strategy, involving attempts to positively reframe the end of the relationship and disassociate from the former partner. The least frequently adopted main strategy was “Sex and substances,” involving substance use (such as alcohol) and casual sex encounters to cope with the end of a relationship. Furthermore, significant main effects of sex and age were observed for several of the identified strategies.

Intimate relationships typically do not last a lifetime, as illustrated by the high rate of divorce (Raley & Sweeney, 2020). For instance, in Greece in 2020 there were 38 divorces for every 100 marriages (Eurostat). Unforeseen circumstances or aging may also result in the death of an intimate partner. Consequently, people are likely to experience a situation where an intimate relationship they wish to continue ends. The termination of such relationships usually involves intense emotional pain (Apostolou et al., 2025) that individuals need to find ways to manage. The aim of the present study is to identify the strategies people employ to cope with the end of a desirable intimate relationship. We will begin by discussing the evolutionary reasons why the end of an intimate relationship is likely to cause emotional pain, and we will then proceed to discuss possible strategies for coping with that pain.

## Emotional Pain and the End of a Desirable Relationship

Children require considerable, reliable, and prolonged parental investment to reach sexual maturity, which is difficult for a single parent to provide (Lancaster & Lancaster, 1987). Moreover, an intimate partner constitutes a critical source of emotional, material, and physical support and protection (Apostolou et al., 2023). As a result, forming a long-term intimate relationship can confer substantial survival, reproductive, and fitness benefits to the parties involved. These benefits translate into strong selection pressures shaping the human mind to seek the formation of such relationships. Accordingly, across all human societies, individuals establish lasting intimate relationships, commonly identified as marriage (Coontz, 2005).

Nevertheless, an intimate relationship that increases fitness for one partner might not necessarily do so for the other. For instance, individuals may experience an increase in their mate value (i.e., how much they worth as mates), and might thus attract intimate partners of higher quality than their current partner. In such cases, the partner who perceives a fitness loss from staying in the relationship is likely to terminate it. Additionally, fitness-increasing relationships may cease due to the death of an intimate partner. Consequently, people may find themselves in circumstances where an intimate relationship they interpret as desirable—due to considerable fitness benefits—comes to an end.

When this happens, individuals generally experience intense negative emotions, including anger, sadness, and despair. In particular, emotions are best understood as adaptations that motivate behaviors increasing one's fitness (Nesse, 2019; Tooby & Cosmides, 2008). Specifically, engaging in fitness-increasing behaviors or encountering fitness-increasing situations typically triggers positive emotions that encourage individuals to continue such behaviors and seek similar circumstances in the future. Conversely, engaging in fitness-decreasing behaviors or encountering such situations triggers negative emotions, motivating individuals to take corrective actions and to avoid similar situations in the future. Accordingly, fitness losses resulting from the end of a beneficial relationship would trigger strong negative emotions, such as sadness and despair, motivating corrective behaviors, including reflecting on mistakes to avoid repetition. These negative emotions can also have a lasting psychological impact (“scarring effect”): When searching for new mates, people recall past emotional pain and consequently become more selective to prevent experiencing these emotions again (Apostolou et al., 2024). Furthermore, this emotional memory assists individuals currently in a fitness-enhancing relationship to make preventive corrections, ensuring their partners do not leave and thereby avoiding renewed suffering.

Empirical evidence aligns with this theoretical framework. One study recruited a sample of 442 Greek-speaking participants and investigated how they would respond to the termination of an intimate relationship they wished to continue (Apostolou et al., 2024). Results indicated the most frequent reaction was experiencing negative emotions such as sadness and despair. In total, both theoretical reasoning and empirical findings underline that the end of a desirable intimate relationship results in substantial emotional pain. This raises the question regarding how individuals cope with this emotional pain, which we address in the following sections.

## Dealing With the End of a Relationship

Apart from being unpleasant, the emotional pain associated with the end of a desirable intimate relationship may disrupt other fitness-increasing activities, such as working to secure subsistence (see also Nesse, 2019). Consequently, individuals face the challenge of managing emotional pain so that it does not interfere substantially with daily functioning. Still, one can ask why have evolutionary selection pressures have not adjusted emotional mechanisms to produce negative emotions strong enough to motivate corrective actions and create a scarring effect, yet moderate enough to avoid considerably disrupting individuals’ lives?

A plausible explanation lies in the intensity required for such emotions to serve their adaptive function effectively. If negative emotions were mild enough to be relatively nondisruptive, they might not sufficiently motivate the corrective actions or the necessary caution when selecting future mates. Nor would they compel individuals to take preventive measures to preserve future beneficial relationships. Therefore, experiencing strong emotional pain after a desirable intimate relationship ends could be adaptive—as long as individuals simultaneously employ strategies to keep those negative emotions manageable, thus minimizing disruption in other aspects of their lives. In this manner, the corrective actions and scarring effects occur, while fitness penalties from excessive emotional disruption are minimized.

While we have emphasized here the scenario in which individuals experience a decrease in fitness following the end of a relationship, similar emotional pain arises from other scenarios, such as illness, death of a relative, or loss of friendship. Observing these recurring sources of emotional pain, selection pressures likely have favored the evolution of general strategies enabling individuals to manage intense emotional pain effectively across diverse situations. Still, if a fitness loss scenario has been recurrent in human evolution, selection forces would have forged a capacity to employ more specialized strategies that would enable better handling of the specific issue. Thus, we hypothesize that people employ a variety of strategies—some general and others more specialized—to cope specifically with the termination of desirable intimate relationships. These strategies would primarily aim at alleviating the emotional pain caused by relationship dissolution. We discuss some possible strategies next.

Emotional mechanisms generate negative emotions in response to fitness-decreasing situations, and positive emotions in response to fitness-increasing ones. Therefore, one likely coping strategy involves engaging in activities that enhance fitness and thereby mitigate the emotional pain following the dissolution of a relationship. For instance, investing in self-improvement activities such as efforts to develop career success, would improve chances to attract higher-quality mates and could bring thus positive emotions and provide relief from emotional distress. Furthermore, humans are inherently social and have evolved to live in groups (Ward & Webster, 2016). Thus, when individuals face difficulties, seeking social and emotional support from others typically helps alleviate distress (Thoits, 2011). Consequently, a coping strategy following the end of a relationship likely involves seeking support from friends and family members. The existing research literature offers additional insight into potential coping mechanisms individuals use after relationship dissolution.

## Current Literature

Extensive research has investigated how individuals cope with stressful life events. In particular, Lazarus and Folkman (1984) proposed the transactional model, defining coping as a dynamic interaction between the individual and the environment, emphasizing subjective appraisal in determining what constitutes stress and appropriate coping responses. They categorized coping approaches into two interrelated categories: Problem-focused coping, aimed directly at managing or altering the stress source, and emotion-focused coping, aimed at regulating emotional responses to stressful situations (Folkman & Moskowitz, 2004; Lazarus & Folkman, 1984). Building upon this conceptualization, Carver et al. (1989) introduced the COPE Inventory, a psychometric measure that identifies fourteen coping strategies such as active coping, planning, acceptance, positive reinterpretation, emotional venting, seeking social support, mental disengagement, and humor. Beyond the binary categorization (problem-focused vs. emotion-focused), subsequent research has highlighted coping strategies oriented toward meaning-making and positive reappraisal (for a review, see Folkman & Moskowitz, 2004).

The termination of a desirable intimate relationship clearly constitutes a stressful experience; thus, strategies identified in the stress-coping literature are relevant. Nevertheless, as discussed earlier, the end of a desirable intimate relationship represents a domain-specific and recurrent scenario in human evolutionary history. Consequently, individuals may employ specialized coping strategies tailored specifically to relationship dissolution. There is, therefore, a need for research specifically examining how people manage the end of desirable intimate relationships. To address this issue, Perilloux and Buss (2008) recruited a sample of 199 students in the United States who reported experiencing at least one romantic breakup, asking them to rate 20 different coping strategies. Some strategies participants rated included substance use, threatening the ex-partner, demonstrating affection to someone else, avoiding the ex-partner, and threatening suicide.

More recently, another study identified three main reactions following the end of an intimate relationship (Apostolou et al., 2024). In the first one, “Accept and forget,” individuals attempt to accept the situation, cease contact entirely with their ex-partner, and occupy themselves with other thoughts and activities. In the second one, “Sadness and depression,” people experience intense negative emotions, such as deep sadness and depression, and typically seek professional psychological support. In the third one, “Physical and psychological aggression,” individuals indicated they would become angry and aggressive toward the ex-partner and might seek revenge through casual sexual encounters (“revenge sex”).

## The Present Study

Although substantial research addresses general stress-coping strategies, relatively limited research has focused specifically on how individuals deal with the termination of desirable romantic relationships. Apostolou et al. (2024) primarily focused on individuals’ emotional reactions following breakups. While these reactions partially imply coping strategies, they primarily constitute reactions rather than explicit coping strategies. For example, frequently reported reactions were sadness and grief—emotional states rather than ways to deal with these states. Similarly, Perilloux and Buss (2008) nominated some specific coping acts than empirically deriving strategies directly from participants. Additionally, their research did not utilize data-reduction methods, leaving it unclear whether these coping acts represented distinct strategies or diverse expressions of a smaller set of underlying strategies. Furthermore, their study did not adequately distinguish between reactions to breakups (such as threatening suicide or the ex-partner), and genuine coping strategies meant to reduce emotional pain.

Overall, existing literature lacks a comprehensive account of the specific strategies individuals employ to manage the end of desirable romantic relationships. The current research aims to fill this gap to our knowledge, by addressing the limitations of previous research: We aimed to compile a comprehensive list of acts and, using data-reduction techniques, classify them into broader strategies. In more detail, we employed a mixed-methods approach. Specifically, in Study 1, qualitative research methods were used to identify the different acts individuals employ to cope with the end of desirable intimate relationships. Subsequently, Study 2 employed quantitative research methods to classify these acts into broader, overarching strategies. Our hypothesis is that coping strategies are selected for effectively alleviating emotional pain. We discussed several possible strategies above, such as seeking support from friends and family. However, because the phenomenon is complex and research in this area is limited, we designed our study to be exploratory and therefore did not test hypotheses about which specific coping strategies might emerge. Furthermore, we investigated the effects of sex, age, and relationship status on individuals’ willingness to use these identified strategies, although no specific directional predictions were made.

## Study 1

### Method

#### Participants

The research was carried out online at a private university located in the Republic of Cyprus, following approval from the university's ethics committee. The study complies with APA ethical standards in the treatment of the sample. Participants were recruited through social media platforms, including Facebook and Instagram, specifically aiming to reach individuals living in Greece and the Republic of Cyprus. Further distribution of the survey link involved sharing with students and colleagues, who were also asked to complete and forward the questionnaire to others. Participation criteria required individuals to be at least 18 years old and to have experienced the end of an intimate relationship in the past; no compensation was offered for participation. The sample included 118 Greek-speaking participants (65 women and 53 men). Women's mean age was 36.1 years (*SD* = 10.0), and men's mean age was 36.6 years (*SD* = 9.2). In terms of relationship status, 37.3% reported being single, 33.9% were in a relationship, 20.3% were married, and 8.5% indicated their marital status as “other.”

#### Materials

At the beginning of the survey, participants were asked to indicate whether they had experienced the end of an intimate relationship (Yes/No). Participants who answered “No” were thanked for their interest, while those who answered “Yes” were directed to the survey. The questionnaire consisted of two sections. In the first section, participants responded to the following task: “Romantic relationships in which people are emotionally committed and want to continue often end for reasons beyond their control. Write down some ways in which you would try to deal with the termination of such a relationship”: The second section gathered demographic information, including biological sex (man, woman, other), age, and relationship status.

### Data Analysis and Results

The data were analyzed in the following way: Two independent graduate students (one female and one male) were assigned to sort the collected responses into broader categories. Similar responses were grouped into existing categories, whereas responses that differed substantially led to the creation of new categories. Unclear or ambiguous answers were excluded from analysis. After approximately 30% of the data had been categorized, the two coders compared their initial results. At this stage, there was an 84% agreement between the two coders. Differences in categorization were addressed and resolved through discussion with one of the authors until complete agreement was reached. Subsequently, the coders proceeded to classify the remaining responses. Overall, 84 acts were identified and are listed in Table 1 and in the Appendix.

**Table 1.** Acts and Strategies for Coping With the End of a Desirable Intimate Relationship.

Strategies acts	Factor loadings	Cronbach's α
Focus on myself		.87
I would focus on myself	.553	
I would spend time with myself	.544	
I would focus on self-improvement	.490	
I would focus on my goals	.414	
I would do things I enjoy	.396	
I would focus on other areas of my life	.339	
Reconciliation attempts		.56
I would focus on finding ways to win him/her back	.900	
I would try to win him/her back	.873	
I would try to reach out to him/her	.825	
I would try to accept it	−.378	
Disassociation strategies		.84
I would throw away things that remind me of him/her (e.g., gifts, clothes)	.775	
I would throw away/delete his/her photos	.734	
I would erase his/her phone number	.718	
I would destroy his/her things	.613	
I would block him/her on social media	.598	
I would cut off all communication with him/her	.448	
I would distance myself from anything related to him/her	.363	
I would try to put any thoughts of him/her out of my mind	.328	
Understand what went wrong		.85
I will try to think about the reasons why the relationship ended	−.829	
I will try to understand the reasons why the relationship ended	−.798	
I will try to process the events and understand the real reason for ending this relationship	−.762	
I will try to understand what went wrong	−.668	
I will try to identify the mistakes I made, so that I don't repeat them in the future	−.598	
I will try to rationalize the reasons that led to the relationship ending	−.468	
Seek new relationships		.81
I would seek to have sex with someone else	−.841	
I would have casual sex	−.786	
I would start dating	−.639	
I would start using dating apps	−.603	
I would seek to start another relationship soon	−.480	
Social withdrawal		.81
I would isolate myself	.917	
I would shut myself off	.826	
I would not talk to anyone	.561	
Seek professional help		.95
I would seek psychological support from a professional	−.962	
I would go to a psychologist	−.952	
I would undergo psychotherapy	−.877	
Positive reframing		.87
I would focus on the hope that I will find someone better	−.709	
I would try to think that I deserve someone better	−.707	
I would try to think that I can find someone better	−.671	
I would try to think that this partner is not right for me	−.611	
I would try to think that since the relationship is over, maybe it wasn't worth it after all	−.567	
I would try to think that this outcome is better for me	−.543	
I would think that it was meant to be	−.485	
I would make a list of his/her negatives to realize how wrong he/she was for me	−.444	
I would try to forget him/her	−.327	
Work therapy		.68
I would dedicate myself to my work/studies	.776	
I would focus on my professional goals	.744	
I would work overtime	.376	
I would pursue a postgraduate degree/postgraduate training	.337	
Family support-seeking		.83
I would ask for advice on how to deal with it from relatives	.792	
I would seek psychological support from relatives (e.g., parents, siblings, cousins)	.783	
I would discuss ending the relationship with my family (e.g., parents, siblings, cousins)	.718	
I would spend more time with relatives (e.g., parents, siblings, cousins)	.648	
Keep myself busy		.76
I would try to keep my mind off of what is going on	.759	
I would keep myself busy with as many things as I can so that I don't have time to think about it	.717	
I would do things to take my mind off it	.703	
I would spend more time with my friends	.311	
Improve looks		.79
I would make changes to my appearance	−.868	
I would improve my looks	−.761	
Friends support-seeking		.78
I would ask friends for advice on how to deal with it	.783	
I would ask friends for psychological support	.694	
I would discuss ending the relationship with friends	.637	
I would discuss ending the relationship with an opposite sex friend	.460	
Start a new activity/hobby		.73
I would start an activity/hobby	.538	
I would seek to engage in a creative/artistic activity	.477	
I would try to get more in touch with nature (e.g., walks in the forest/park)	.432	
I would find new interests	.417	
Use substances		.58
I would use drugs	.623	
I would consume alcohol	.562	
Acceptance of emotions		.68
I would let myself experience unpleasant emotions	−.756	
I would let myself get angry	−.564	
I would let myself cry	−.560	

**Table 2.** Factor Loadings for Main Strategies for Coping With the End of a Desirable Intimate Relationship.

Main strategies	Factor loadings
Transfer focus to different things	
Focus on myself	.770
Start a new activity/hobby	.710
Work therapy	.689
Keep myself busy	.477
Improve looks	.447
Seek support	
Friends support-seeking	.753
Acceptance of emotions	.484
Understand what went wrong	.446
Family support-seeking	.423
Seek professional help	.325
Sex and substances	
Seek new relationships	.671
Use substances	.480
Social withdrawal	
Social withdrawal	−.695
Disassociation and positive reframing	
Disassociation strategies	.879
Positive reframing	.518
Reconciliation attempts	−.322

## Study 2

### Method

#### Participants

Participants were recruited in Greece and in the Republic of Cyprus using the same procedure described in Study 1. The study complies with APA ethical standards in the treatment of the sample. A total of 528 Greek-speaking individuals took part, consisting of 284 women, 218 men, five participants who identified their sex as “other,” and one who did not answer the question about biological sex. The mean age for women was 30.9 years (*SD* = 12.3), and for men, it was 31.1 years (*SD* = 13.1). Additionally, 51.4% of participants were single, 27.9% were in a relationship, 14% were married, and 6.7% reported their relationship status as “other.” All data are available here: https://osf.io/u23hc/?view_only = 8fbd6ac414364f179ec452d151a06631.

### Materials

Initially, participants were asked to indicate whether they had experienced the end of an intimate relationship in the past (Yes/No). Participants who answered “No” were thanked for their interest, while those who answered “Yes” were directed to the main survey. The survey, conducted in Greek, consisted of two sections. In the first section, participants were given the following scenario: “Romantic relationships in which people are emotionally committed and want to continue often end for reasons beyond their control. Indicate how likely you are to do each of the following in order to deal with the end of such a relationship”: Participants then rated the 84 acts identified in Study 1 using a 5-point Likert scale, where 1 represented “*not at all likely to do it*” and 5 represented “*very likely to do it*.” The order in which the acts were presented was randomized for each participant. The second section gathered demographic information, including biological sex, age, and relationship status.

### Data Analysis

To classify the various acts into broader categories, we used exploratory factor analysis with the principal axis factoring method for factor extraction and direct oblimin for rotation. The decision on the number of extracted factors was guided by the Kaiser criterion, retaining all factors with an eigenvalue of at least one. Although the Kaiser criterion has been criticized for retaining too many factors, other techniques—such as the scree plot and parallel analysis—have been criticized for retaining too few (Field, 2024). Because this is the first study to classify different acts into broader strategies and we do not want to overlook any important strategies, we adopted the Kaiser criterion. Principal axis factoring analysis was performed twice: First on the acts for dealing with the breakup to classify them into broader factors or strategies, and second on the extracted factors to classify them into even broader domains or main strategies. To identify significant effects, we conducted a series of MANCOVA tests, entering the strategies composing a main strategy as dependent variables. In this analysis, sex and relationship status were included as categorical independent variables, and age was included as a continuous independent variable. The procedure was repeated for each extracted main strategy. The statistical analysis was performed using the SPSS version 28 software package.

## Results

### Factor Structure

#### First Order

The Kaiser–Meyer–Olkin (KMO) statistic indicated that our sample was suitable for exploratory factor analysis (KMO = .87). To determine which items to retain in each factor, we utilized a factor loading cutoff of .30 (Field, 2024). The initial analysis produced a factor structure where several items loaded below this threshold. Consequently, we removed the item with the lowest loading and re-ran the analysis, repeating this procedure iteratively until all remaining items had loadings of at least .30. In total, 13 items were removed, and are listed in the Appendix. These items included acts such as turning to religion, overeating, travelling, moving to a different city/country, and taking psychotropic drugs. The remaining 71 items were grouped into 16 distinct strategies for coping with the end of a desirable intimate relationship (presented in Table 1). Internal consistency for these strategies, assessed using Cronbach's alpha, ranged from .58 to .95, with an overall mean of .78 (Table 1).

The first strategy identified was “Focus on myself,” in which participants indicated that they would cope with the end of the relationship by concentrating on personal improvement, pursuing personal goals, and doing enjoyable activities. The second identified strategy was “Reconciliation attempts,” involving efforts aimed at winning the ex-partner back. Additionally, participants indicated they would engage in “Disassociation strategies,” which included throwing away or destroying items associated with their former partner, deleting contact information, erasing shared photographs, and blocking the ex-partner on social media. Another strategy, labeled “Understand what went wrong,” included actively attempting to analyze and understand why the relationship failed.

In the “Seek new relationships” strategy, participants expressed willingness to engage in casual sexual encounters and actively pursued opportunities for dating or acquiring a new partner, sometimes via dating applications. Conversely, the “Social withdrawal” strategy was characterized by the avoidance of social interactions and isolation from others. Another identified coping mechanism was termed “Seek professional help,” where participants indicated that they would consult mental-health professionals, such as psychologists. Additionally, participants endorsed the “Positive reframing” strategy, which involved attempts to reinterpret the breakup positively—such as thinking the former partner was not an adequate match, and focusing optimistically on finding a better partner in the future. Another strategy emerged as “Work therapy,” involving dedicating extra time and attention to work and professional achievements.

In the “Family support-seeking” strategy, individuals would seek emotional and practical support from family members, including parents, siblings, and extended family. Another strategy, “Keep myself busy,” involved occupying oneself with numerous activities to distract from thoughts about the breakup. Moreover, participants indicated they would use a strategy labeled “Improve looks,” investing in enhancing their physical appearance. Additionally, the “Friends support-seeking” strategy involved seeking emotional support and advice from friends. In the “Start a new activity/hobby” strategy, individuals would initiate new hobbies or activities, such as spending more time outdoors or taking nature walks. Another identified coping strategy was “Use substances,” involving the use of substances such as alcohol or drugs to mitigate the emotional distress caused by the breakup. Lastly, participants identified the strategy of “Acceptance of emotions,” involving allowing themselves to experience and express the negative emotions associated with relationship dissolution, such as crying or feeling anger.

#### Second Order

We created 16 new variables by averaging the items comprising each of the identified strategies described above. Exploratory factor analysis conducted on these new variables revealed five domains, or main strategies (Table 2). The first main strategy identified was “Transfer focus to different things,” in which individuals attempted to divert attention from the dissolution of the relationship by keeping busy, focusing on self-development, dedicating themselves to professional goals, and improving their appearance. The second main strategy, “Seek support,” reflected participants’ preference for obtaining support from family, friends, and mental health professionals to manage the end of the relationship and gain a deeper understanding of why it occurred.

The third main strategy, “Sex and substances,” involved coping with relationship dissolution through casual sexual encounters or by consuming substances, such as drugs or alcohol. The fourth main strategy identified, “Social withdrawal,” involved participants isolating themselves and withdrawing from social contact. Lastly, the fifth main strategy, “Disassociation and positive reframing,” encompassed coping behaviors where participants sought to frame the breakup positively, asserting that ending the relationship was beneficial overall. This approach featured disassociation strategies and positive reframing. Higher willingness to employ this strategy was associated with lower willingness to attempt to win back the ex-partner.

We calculated the means and standard deviations for each strategy and main strategy, placing them in hierarchical order as presented in Table 3. Additionally, for each main strategy, we calculated the percentage of participants who had a mean score higher than “3.” Given our one-to-five rating scale, this percentage reflects how many participants expressed a relatively high willingness to use a particular strategy. As shown in Table 3, the main strategy most likely to be used was “Transfer focus to different things,” with approximately 85% of participants expressing a high willingness to employ it. Both “Social withdrawal” and “Seek support” were also likely to be used main strategies. Conversely, “Sex and substances” was the least likely to be used main strategy, with only about 15% of participants indicating a high likelihood to utilize it. Considering individual strategies, “Understand what went wrong” received the highest mean score, closely followed by “Focus on myself.” Other frequently preferred strategies included “Keep myself busy,” “Acceptance of emotions,” and “Friends support-seeking.” At the opposite end, participants indicated that “Use substances” and “Seek new relationships” were the strategies they would be least likely to employ.

**Table 3.** Means, Likelihood of Adoption, and Main Effects of Sex, Age, and Relationship Status on the Identified Strategies.

	Overall				Sex		Age		Relationship status	
Main strategies	*M* (*SD*)	%	Women	Men	*p*	η_p_*^2^*	*p*	η_p_*^2^*	*p*	η_p_*^2^*
Transfer focus to different things	3.66 (0.64)	84.6	3.76 (0.63)	3.52 (0.64)	.041	.020	.005	.035	.039	.018
Focus on myself	4.10 (0.77)		4.16 (0.75)	4.00 (0.78)	.009	.020	.096	.006	.626	.004
Keep myself busy	4.02 (0.80)		4.11 (0.78)	3.88 (0.82)	.053	.012	(−) .040	.009	.275	.008
Start a new activity/hobby	3.53 (0.91)		3.66 (0.88)	3.35 (0.93)	.006	.022	.609	.001	.739	.003
Improve looks	3.53 (1.11)		3.64 (1.12)	3.36 (1.08)	.010	.019	.496	.001	.495	.005
Work therapy	3.15 (0.89)		3.24 (0.88)	3.03 (0.88)	.007	.021	(−) .004	.017	.053	.016
Social withdrawal	3.58 (1.32)	56.6	3.77 (1.26)	3.31 (1.34)	.001	.027	.351	.002	.443	.006
Social withdrawal										
Seek support	3.35 (0.66)	72.6	3.54 (0.58)	3.11 (0.68)	<.001	.053	<.001	.065	.192	.014
Understand what went wrong	4.11 (0.74)		4.14 (0.64)	4.09 (0.83)	.105	.010	.461	.001	.644	.004
Acceptance of emotions	3.97 (0.91)		4.23 (0.73)	3.63 (1.00)	<.001	.054	.587	.001	.090	.014
Friends support-seeking	3.54 (1.05)		3.68 (1.02)	3.34 (1.07)	.045	.013	(−) .001	.023	.570	.004
Seek professional help	2.71 (1.44)		3.00 (1.45)	2.33 (1.34)	.001	.028	(+) < .001	.028	.455	.006
Family support-seeking	2.43 (1.12)		2.64 (1.15)	2.15 (1.03)	<.001	.042	.139	.005	.138	.012
Disassociation and positive reframing	2.93 (0.54)	43.9	2.97 (0.52)	2.88 (0.56)	.030	.015	.605	.004	.582	.005
Positive reframing	3.27 (0.91)		3.39 (0.89)	3.11 (0.91)	.002	.027	.702	.000	.488	.005
Reconciliation attempts	2.85 (0.83)		2.80 (0.80)	2.92 (0.85)	.466	.003	.305	.002	.608	.004
Disassociation strategies	2.68 (0.91)		2.72 (0.88)	2.62 (0.97)	.368	.004	.560	.001	.279	.008
Sex and substances	2.10 (0.82)	14.7	1.85 (0.75)	2.41 (0.82)	<.001	.038	<.001	.045	.112	.011
Seek new relationships	2.18 (0.98)		1.89 (0.82)	2.56 (1.03)	<.001	.064	.679	.000	.559	.004
Use substances	2.00 (1.08)		1.81 (0.99)	2.25 (1.15)	.001	.029	(−) < .001	.040	.048	.017

Furthermore, we calculated that 5.3% of participants did not report a high willingness to use any main strategy. Conversely, 9.9% of participants indicated a relatively high willingness for one main strategy, 23.3% for two, 32.3% for three, 24.9% for four, and 3.8% for all five main strategies. Accordingly, the majority of participants (84.3%) demonstrated relatively high willingness to utilize more than one main strategy.

## Significant Effects of Sex, Age, and Relationship Status

We conducted five MANCOVA tests; therefore, to decrease the probability of Type I error, we applied a Bonferroni correction for alpha inflation, adjusting the significance level to .01 (.05/5). Consequently, effects above this adjusted value should not be considered statistically significant. As illustrated in Table 3, there was a significant main effect of sex for three out of five main strategies—and indeed for all strategies, if Bonferroni correction was not applied. Effect-size measures indicated that the largest sex difference occurred for the “Seek support” strategy, with women scoring significantly higher than men, followed by “Sex and substances,” for which men reported significantly higher scores than women. In most of the cases, the effect sizes were small, indicating a considerable overlap between the two sexes.

Regarding participants’ age, significant main effects emerged for the “Sex and substances” and “Transfer focus to different things” strategies. Specifically, the age effect within “Sex and substances” occurred predominantly within the individual strategy “Use substances,” whereas the age effect identified within “Transfer focus to different things” occurred within the individual strategies “Keep myself busy” and “Work therapy.” In each instance, the regression coefficients were negative, signifying that older participants reported lower scores than younger participants. Generally, in most cases the effect sizes were very small, indicating a limited influence of age on the identified strategies. Finally, there was no statistically significant main effect of relationship status on any of the identified strategies.

## Discussion

In the present research, we investigated the various strategies individuals employ to deal with the end of a desirable intimate relationship. A two-level structure emerged, comprising 84 different acts categorized into 16 strategies, which were further grouped into five main strategies. The most frequently endorsed main strategy was “Transfer focus to different things,” which included coping strategies such as “Focus on myself” and “Keep myself busy.” The “Seek support” main strategy, which involved obtaining support from friends, family, and professionals, as well as the “Social withdrawal,” was also commonly adopted. A considerable proportion of participants indicated willingness to utilize the “Disassociation and positive reframing “main strategy, which entailed attempts to positively reframe the end of the relationship and disassociate from the former partner. The least frequently endorsed main strategy was labeled “Sex and substances,” which involved consuming alcohol or drugs and pursuing casual sexual relationships as strategies to cope with the dissolution of an intimate relationship. Additionally, significant main effects of sex and age were found for several strategies.

We hypothesized that individuals possess a range of coping strategies enabling them to manage effectively the end of an intimate relationship by easing associated emotional pain. With the exception of “Social withdrawal,” the identified main strategies generally aligned with this hypothesis. Specifically, the most likely to be used main strategy, “Transfer focus to different things,” involved individuals diverting their attention toward self-improvement activities, such as career advancement, new hobbies, personal goals, or enhancing physical appearance. These activities could yield fitness-related benefits—for example, improved appearance may facilitate attracting new intimate partners—and thus generate positive emotions that mitigate negative ones arising from the stressor. Apart from fostering positive emotions, shifting one's attention away from emotional pain likely reduces distress. Accordingly, approximately 85% of participants expressed high willingness to employ this strategy.

Humans, as inherently social beings, typically seek comfort and advice from others. Accordingly, participants showed strong inclination toward seeking emotional support from personal networks, including friends, family members, and mental-health professionals (i.e., “Seek support”). Discussing issues with others helps individuals better understand relationship failures and avoid repeating mistakes in the future. Another dimension of this approach involved openly expressing (potentially to others) their negative emotions, such as sadness or crying, to alleviate emotional distress. This was a commonly endorsed strategy, with nearly 73% of participants reporting a high willingness to use it.

The end of a beneficial intimate relationship generates considerable losses in fitness and accompanying intense negative emotions. Nonetheless, individuals might partially ease this distress if they can successfully persuade themselves that the ended relationship was not beneficial in the first place. Thus, within the “Disassociation and positive reframing” main strategy, participants attempted to convince themselves that the former partner was not a suitable match and that they deserved someone better. Another aspect of this strategy—disassociating oneself from reminders of the partner—also mitigates distress; confronting frequent reminders might otherwise intensify emotional pain. However, if people fail in their attempts at positive reframing, they may instead attempt reconciliation, explaining the negative loading of the “Reconciliation attempts” strategy onto this main strategy. The “Disassociation and positive reframing “strategy was moderately popular, with about 44% of participants indicating a strong willingness to use it.

The “Sex and substances” main strategy involved behaviors such as pursuing casual sexual encounters and consuming substances such as alcohol or drugs. Reentering the mating market and engaging in casual relationships might temporarily distract from emotional distress or inspire hope for future relationships. Additionally, substance use temporarily suppresses emotional pain, acting as an emotional painkiller (Khantzian, 1997). Yet, these strategies carry considerable risks and negative consequences, such as contracting sexually transmitted diseases (Fielder et al., 2014) or developing substance-use disorders (Lopez-Quintero et al., 2011). Thus, it is not surprising this was the least preferred strategy, endorsed highly by only about 15% of participants.

More than half of the participants reported a strong willingness to adopt the “social withdrawal” strategy. One explanation is that withdrawal offers several coping benefits, including space for retrospection: Individuals gain time to reflect on the breakup and draw lessons for future choices. Social withdrawal also limits exposure to social comparison (e.g., being around others who are in relationships) and unsolicited advice, both of which can heighten distress. In addition, it shields individuals from contact with an ex-partner or revisiting places that might trigger rumination or painful memories. Nevertheless, withdrawal can intensify feelings such as loneliness and sadness, so people may rely on it only for a limited period.

We argue that the end of an intimate relationship tends to generate considerable emotional pain that can reduce fitness if left unmanaged. Because breakups almost certainly occurred in ancestral societies and were similarly distressing, natural selection should have favored strategies that help individuals cope with such pain. These strategies are therefore expected to be broadly universal, present across historical periods and cultures. For instance, given humans’ fundamentally social nature, seeking emotional support after a breakup should appear consistently in diverse cultural settings. At the same time, selection has favored cognitive plasticity, enabling people to tailor their behavior—including coping strategies—to the cultural context they inhabit. Seeking comfort from others might involve consulting a psychologist in contemporary Western societies, whereas in preindustrial settings the same role could have been filled by a priest or an elder. We propose that humans share a core set of coping strategies that display only superficial cultural variation, a hypothesis that future cross-cultural research should examine.

A moderate-sized sex difference was identified for the “Seek support” main strategy: Women reported stronger willingness to seek social and professional support following the end of a romantic relationship than men. Possible explanations for this sex difference include women's greater sociability compared to men (Schmitt et al., 2008) and women's higher interest in psychology (Su et al., 2009), potentially making women more comfortable seeking professional psychological help. Conversely, men tend to express greater interest in casual sex (Buss & Schmitt, 2019) and higher levels of substance abuse (Brady & Randall, 1999). Consistent with these patterns, we identified a small-to-moderate effect of sex for the “Sex and substances” strategy, with men expressing greater willingness than women to pursue casual sex and substance use. Additionally, women showed slightly greater willingness than men to adopt “Social withdrawal,” but we do not currently possess a hypothesis to explain this finding.

A modest age effect emerged in relation to seeking social support. Specifically, older participants reported lower willingness than younger participants to seek support from friends, perhaps because younger adults attribute more importance to friendship than older adults (Hruschka, 2010). Conversely, older participants expressed greater willingness to seek professional help following the end of a relationship, which may reflect older individuals’ greater financial resources or broader awareness of psychological services. Finally, one might expect that individuals currently in an intimate relationship would report a greater willingness to adopt any of the identified strategies because they can more readily empathize with coping with a breakup than single individuals. Yet, relationship status showed no significant main effects on any of the identified strategies. A plausible explanation is that every participant in our sample had previously experienced the end of an intimate relationship and could therefore empathize with the scenario regardless of their current relationship status.

Carver et al. (1989) identified 14 general strategies for coping with stressors. Considering relationship dissolution as a stressful event, some identified strategies overlap directly with those in the present study, such as seeking social support (instrumental and emotional), positive reinterpretation and growth, emotional venting, mental disengagement, and alcohol-drug disengagement. Nevertheless, strategies from the Carver et al. instrument such as active coping, planning, and suppression of competing activities were absent from our results. An explanation for this discrepancy is that the Carver et al. instrument addresses general stressors, emphasizing direct resolution of problems through active coping. In contrast, our study explicitly addresses a permanent loss (the end of an intimate relationship), which requires emotional coping rather than active mitigation of a temporary stressor.

Similarly, Perilloux and Buss (2008) nominated 20 coping strategies for breakup scenarios. Some overlap exists—for example, discussing the breakup, consuming alcohol heavily, and showing affection to other partners—but overall, there was limited congruence with our findings. Their list included items such as crying, threatening suicide, or partner abuse that reflect immediate reactions rather than deliberate coping strategies to ease emotional pain. Apostolou et al. (2025) specifically investigated reactions rather than coping strategies; thus, their structures differed considerably from ours (e.g., feeling sad or angry). Nevertheless, some reactions they identified (e.g., occupying one's mind or consulting a psychologist) align partially with our identified strategies. Overall, the differences in the findings of previous studies with our own underline the importance of distinguishing deliberate coping strategies from initial reactions, and the need for appropriately employing data-reduction techniques.

We also found that most participants indicated willingness to use multiple coping strategies simultaneously. Utilizing multiple strategies likely achieves more effective emotional easing than relying on a single approach. As an illustrative example, emotional pain following the termination of a desirable intimate relationship would likely be better reduced by utilizing a combination of positive reframing, shifting attention to unrelated activities, and seeking social support, rather than relying solely on reframing. Future research should assess explicitly the effectiveness of these individual and combined coping approaches. Additionally, the effectiveness—and thus strategy selection—is likely influenced by the specific circumstances that the relationship ended. For example, in cases where a partner dies, strategies such as positive reframing and seeking casual sex are unlikely to be used, whereas in scenarios involving partner infidelity these strategies might gain prominence.

In the present study, we identified several strategies for coping with the end of a valued intimate relationship. A logical next step is to assess the effectiveness of each strategy. Future longitudinal research could follow individuals who are currently in relationships, document the strategies they adopt when a breakup occurs, and evaluate the outcomes. For instance, researchers might measure emotional well-being (e.g., happiness, loneliness) at the time of the breakup and track changes over time as a function of the strategy used. Our findings also suggest that people often combine strategies; some combinations may prove more effective than others, a possibility that warrants further investigation.

More theoretical and empirical work is necessary to understand how the strategies that people use to cope with fitness-decreasing events—particularly the end of an intimate relationship—ultimately function. We argue that an effective combination of strategies should lead to future fitness gains, such as attracting and retaining a more suitable partner. Specifically, individuals might use “Understand what went wrong” to identify and avoid repeating past mistakes, “Focus on myself” to enhance personal strengths, and “Seek new relationships” to meet prospective mates. Future studies could adopt longitudinal designs to examine the combinations of strategies people choose after the dissolution of a valued intimate relationship and to determine which combinations result in fitness-enhancing outcomes, such as forming a more stable partnership.

One limitation of the present study is reliance on self-report data, potentially introducing response biases. Additionally, nonprobability sampling limits generalizability. Moreover, our research took place within a Greek cultural context, meaning results may not extend directly to other cultural environments. Thus, future cross-cultural studies should attempt to replicate our findings and examine culturally relevant factors affecting strategy use. Additionally, exploratory factor analysis is sample-dependent; replication is needed to confirm the structure we identified. For instance, several items were excluded from the analysis due to their low loading, indicating that some conceptually relevant strategies may have been lost in the process, an issue that replication studies can address. Furthermore, future research should investigate additional predictors of strategy choices beyond sex, age, and relationship status, such as personality traits or prior breakup experiences.

In summary, the present research has identified 84 acts grouped into 16 strategies and further categorized into five main strategies that individuals employ to cope with the end of desirable intimate relationships. We also found differences between sexes and age groups in willingness to use the identified strategies. The complexity identified suggests that further research remains necessary to better understand this phenomenon.

## Appendix

1. I would seek to change my environment (e.g., home, city, country).
2. I would go travelling.
3. I would think that it is not something I can control.
4. I would try to eliminate my negative emotions.
5. I would overeat.
6. I would read self-improvement books.
7. I would get a pet for comfort.
8. I would turn to religion.
9. I would take psychotropic drugs.
10. I would do sports/physical exercise.
11. I would engage in conversation with AI (e.g., ChatGPT).
12. I would go out more with friends.
13. I would write down my feelings in a journal to process them better.
